# Methods of Controlling Microbial Contamination of Food

**DOI:** 10.3390/pathogens14050492

**Published:** 2025-05-16

**Authors:** Renata Urban-Chmiel, Jacek Osek, Kinga Wieczorek

**Affiliations:** 1 Department of Veterinary Prevention and Avian Diseases, Faculty of Veterinary Medicine, University of Life Sciences in Lublin, Głęboka 30, 20-612 Lublin, Poland; 2 Department of Microbiology of Food and Feed, National Veterinary Research Institute, Partyzantów 57, 24-100 Pulawy, Poland; josek@piwet.pulawy.pl (J.O.); kinga.wieczorek@piwet.pulawy.pl (K.W.)

**Keywords:** food preservation, bacteriophages, foodborne diseases, microbiological control, food safety

## Abstract

The rapid growth of world population and increase in living standards have led to an increase in the demand for high-quality, safe food. The Food and Agriculture Organization of the United Nations (FAO) estimates that by 2050 the demand for food will increase by 60%, and production of animal protein will increase by 1.7% a year, with meat production to increase by nearly 70%, dairy products by 55%, and aquaculture by as much as 90%. Microbial contamination of food is a significant problem for the accessibility of safe food which does not pose a threat to the life and health of consumers. Campylobacter, Salmonella, and Yersinia are responsible for thousands of food-borne infections in humans. Currently, numerous programs are being developed to combat pathogenic bacteria in the food supply chain, especially at the primary production stage. These approaches include physical, chemical, biological, and other strategies and methods used to inhibit the bacterial growth of bacteria or completely eliminate the pathogens from the food chain. Therefore, an extremely important goal is to provide safe food and control its quality by eliminating pathogenic and spoilage microorganisms. However, the use of chemicals in food preservation has negative effects for both the consumption values of food and the natural environment. Therefore, it seems absolutely necessary to implement measures utilizing the most environmentally friendly and effective techniques for controlling microbial contamination in food. There is a great need to develop ecological methods in food production which guarantee adequate safety. One of these methods is the use of bacteriophages (bacterial viruses) naturally occurring in the environment. Given the above, the aim of this study was to present the most natural, ecological, and alternative methods of food preservation with regard to the most common foodborne zoonotic bacteria. We also present methods for reducing the occurrence of microbial contamination in food, thus to produce maximally safe food for consumers.

## 1. Introduction

The health status of the human population and its socioeconomic effects are contributing to an increase in efforts to improve food safety and reduce the transmission of foodborne diseases. The FAO suggests that by 2050 the consumption of food will increase to about 60%, and production of animal protein will increase by 1.7% a year, including meat by about 70%, dairy products by 55%, and seafoods up to 90%. Microbial contamination of food is a significant problem for the accessibility of food which is safe for consumers and does not pose a threat to their lives and health [1,2].

Safe production of food free of harmful microbial contaminants posing a potential threat to human health and life is a very important issue all over the world. The World Health Organization has estimated the occurrence of infections caused by foodborne pathogens at about 600 million a year around the world, resulting in very serious illnesses requiring hospitalization and causing high numbers of deaths, estimated at about 420,000 annually. The direct financial costs of medical care and indirect costs as an effect of reduced work productivity may exceed USD 110 billion annually [1].

For these reasons, the production of safe and healthy food is one of the main priority areas of the ‘One Health’ approach with regard to controlling and reducing the transmission of foodborne pathogens [2]. An important problem in this strategy is the common prevalence of pathogens in the environment and many ways of transmission from animals, which are often asymptomatic carriers, to consumers. In EU countries, 251,603 cases of foodborne infections and 1450 foodborne disease outbreaks with 10,894 persons involved were diagnosed in 2023. The vast majority of these infections were due to bacterial agents [3]. Another major threat is the potential transmission of infectious agents from food-producing animals to other animals, crop plants, and the food-processing environment, as well as directly to humans, omitting the food vector [4].

The aim of the present study is to present the most common foodborne zoonoses and methods for reducing the occurrence of microbial contamination of food, and thus to produce maximally safe food for consumers.

## 2. The Most Important Foodborne Zoonoses

According to the zoonoses reports published by the European Food Safety Authority (EFSA) and the European Centre for Disease Prevention and Control (ECDC) (“EU One Health Zoonoses Summary Report”), which present data about the sources of foodborne diseases, the most common disease is campylobacteriosis, which was confirmed in 2022 in 137,107 people from 27 countries of the EU, with an incidence rate of 43.1/100,000 people [5]. As in previous years, campylobacteriosis was most often caused by *C. jejuni* (87.6% of isolates whose species was determined) and much less often by *C. coli* (10.7%); there were also isolated cases of illness caused by *C. fetus* (0.26%), *C. upsaliensis* (0.17%), and *C. lari* (0.12%). The incubation period of campylobacteriosis ranges from 2 to 5 days. The clinical signs mainly affect the alimentary system, like diarrhea, stomachache, and nausea, and usually resolve after a few days. Complications such as joint inflammation or periodic paralysis of the nervous system (Guillain–Barré syndrome) are usually the result of infection by *C. jejuni* [6,7,8]. Infections have resulted in numerous hospitalizations, amounting to 10,551 people in 2022, of which 34 died. The major source of infection was fresh poultry meat.; the process hygiene criterion for Campylobacter in samples of broiler carcasses was established at 1000 cfu/g, hence the limit for the presence of Campylobacter was established as 1000 CFU/g of this fresh meat (Commission Regulation (EC) No. 2073/2005) [9,10]. In 2022, about 17.5% of results exceeded this limit (Commission Regulation (EC) No. 2073/2005) [9,10]. About 17.5% of results exceeded this limit in 2022 [5].

The second most common foodborne disease in the EU was salmonellosis, caused by various serovars of *Salmonella enterica*, especially *S. typhimurium* and *S. enteritidis* [5]. In humans, the disease is most often characterized by fever, stomachache, nausea, and sometimes vomiting. These symptoms are usually mild and often resolve after a few days. In some cases, the body may become dehydrated, and antibiotic and symptomatic treatment is needed. A total of 65,208 salmonellosis cases were confirmed in 2022, with an average incidence rate of 15.3/100,000 inhabitants. The number of these cases was much higher than in the years 2021–2020. Patients were hospitalized in 38.9% of cases, and unfortunately, 81 of them died.

*Salmonella* bacteria occur in most homoeothermic animals, but the most common carriers are various poultry species, especially broilers, but also layer hens and turkeys. Monitoring of broiler breeding flocks in all EU countries showed that in most of these countries, the rate of infection of flocks with serovars *S. enteritidis*, *S. typhimurium* (including the monophasic variant), *S. virchow*, *S. infantis*, and *S. hadar* reached at least 1%—the level which according to legal regulations poses an unacceptable risk [5]. Nevertheless, testing of food of animal origin, including raw poultry meat, showed that it may be contaminated with *Salmonella* at a rate of 7.0% to 8.9%, thus posing a threat to consumer health [5].

Another significant foodborne disease in the EU is yersiniosis, caused by Yersinia enterocolitica (98.7% of prevalence, with most often serotype O3 and to a lesser extent O9 and O8), and sporadically by *Y. pseudotuberculosis* (1.3% of cases) [5]. Infections caused by *Y. enterocolitica* are most common in children, and the typical symptom is diarrhea, often with blood. Older patients may also have stomachache and fever. Symptoms appear 4–7 days after infection and can last up to three weeks or longer. In 2022 in 26 EU countries (no monitoring was conducted in the Netherlands), 7912 people infected with *Yersinia* and presenting disease symptoms were reported (incidence rate of 2.2/100,000 inhabitants), which was a significant increase relative to 2021 (6789 cases). The occurrence of *Yersinia* in domestic animals has only been estimated in a few EU countries, in which a total of 23,705 of these animals were tested, with 0.34% testing positive for *Y. enterocolitica* and 0.32% for *Y. pseudotuberculosis*. Similarly, testing of food has been limited, both ready-to-eat (RTE) and non-RTE, a small percentage of which was found to be contaminated (about 3.5%). Nevertheless, yersiniosis is a significant problem associated with foodborne infections in humans [5].

Much more dangerous than yersiniosis, although less common, are infections caused by verotoxigenic *Escherichia coli* (VTEC), also known as shigatoxigenic *E. coli* (STEC). These bacteria are responsible for intestinal illnesses, commonly presenting as bloody diarrhea, but are also the cause of dangerous complications, such as hemolytic uremic syndrome (HUS), characterized by acute kidney failure and hemolytic anemia [11]. People become infected by eating food contaminated with VTEC bacteria, usually beef and milk, but also water, vegetables, and fruits. Cases in humans are usually sporadic. In previous years, the illness was most often caused by isolates belonging to serotype O157:H7, but recently, especially in the EU countries, VTEC strains belonging to serological groups O26, O157, O80, and O145 have primarily been identified. According to the latest report on zoonoses, in 2022 there were 7117 laboratory-confirmed cases of VTEC infection in 27 EU member countries, with an average incidence rate of 2.1/100,000 inhabitants. According to the report, 38.5% of patients required hospitalization and 28 died, most often children up to the age of 4 years (7 individuals) and people over the age of 65 (12 individuals). HUS was confirmed in 562 patients of all ages, but most in the range of 0–4 years [11].

The carriers of VTEC are various farm animals and wild animals, but most often cattle and small ruminants. In 2022, asymptomatic carriage was noted in 41.5% of cattle and 1.3% of sheep and goats [5]. These bacteria have also been detected in foods of animal origin (beef and milk) as well as plant origin (fruits, vegetables, and juices), secondarily contaminated during production and trade. Bacterial contamination with VTEC also occurs in the case of improperly prepared ready-to-eat products, such as soups, sauces, processed poultry products, and salads [12].

One of the most dangerous foodborne diseases is listeriosis. Cases in humans are due almost exclusively to infection with *Listeria monocytogenes*; among other *Listeria* species, only *L. ivanovii* and *L. seeligeri* can exceptionally be isolated from humans [13,14]. *Listeria* bacteria are widespread in nature, especially in soil, fodder, and water. Infections with these bacteria in adults usually do not cause disease symptoms. However, illness can be a problem in children, older people, or people with a weak immune system, and may cause flu-like symptoms and diarrhea, but also sepsis and meningitis. Infections in pregnant women are a major problem, when the bacteria enter the uterus, which may result in the birth of a sick child or even fetal death [15].

Listeriosis in humans is relatively rare, but has a high mortality rate. In 2022, it was diagnosed in 2738 people in the EU (average incidence rate 0.62/100,000 inhabitants), which was an increase relative to 2021 (2183 cases). A total of 1330 people were hospitalized, which was the highest percentage of foodborne zoonotic diseases. A high number of cases proved fatal (286 people). In the USA, the Centers for Disease Control and Prevention estimate that about 1600 people suffer from invasive *L. monocytogenes* infection each year, with a hospitalization rate of about 94% and about 260 deaths [15,16].

Studies on the occurrence of *L. monocytogenes* in livestock showed the highest prevalence of this bacteria in sheep and goats (5.8%) and cattle (1.2%) [5]. Listeria monocytogenes was also found in foods of animal origin, i.e., ready-to-eat foods such as fish and fish products, processed meat products, and milk and dairy products. Quantitative analyses also showed that some categories of RTE food may contain *L. monocytogenes* in numbers exceeding the acceptable level of cfu/g [5].

## 3. Methods of Reducing Microbial Contamination of Food

Microbial contamination of food poses a significant risk, in many cases leading to death. Ensuring safe food for consumers is one of the priorities of the European Union and other countries around the world. This is achieved in part by producing food from material with a low level of microbes which are pathogenic or potentially pathogenic for humans. Another major and extremely important goal is to ensure food safety and quality control by inactivating pathogenic and spoilage microorganisms [17].

For production of safe food, many programs are developed to combat pathogenic bacteria in the food supply chain, especially at the primary production stage. In accordance with this strategy, the occurrence of *Salmonella* is restricted in poultry flocks, especially in food poultry such as broiler chickens (*Gallus gallus*). According to EU legislation, the level of five *Salmonella* serovars, i.e., *S. enteritidis*, *S. typhimurium* (including monophasic strains), *S. infantis*, *S. virchow*, and *S. hadar*, may not exceed 1% in these flocks (Regulation 2160/2003). Similarly, in the case of meat turkeys, the percentage of birds infected with *S. typhimurium* and/or *S. enteritidis* should not exceed 1% [18]. These control programs include detailed descriptions of both sampling methods and the laboratory analyses and procedures required when *Salmonella* is detected, including biosafety rules, e.g., refraining from transporting, exporting, or selling meat, broiler carcasses, feed, droppings, poultry litter, and other items located where broilers are kept. To ensure consumer safety, microbiological criteria have been established for certain food categories and selected pathogenic bacteria. One of these is the above-mentioned *L. monocytogenes*, for which quantitative or qualitative limits have been defined, i.e., an acceptable level of 100 cfu/g or complete absence in 25 g [9].

In the case of *Salmonella*, the tolerance level is zero, i.e., absence in 25 g. These legal requirements ensure that food meeting these criteria will be safe for consumers.

The development of methods to reduce the occurrence of microbiological contamination in food is crucial to ensuring that products are safe for consumers. Various physical, chemical, and biological methods are used to inhibit bacterial growth or completely eliminate bacteria from food. They are meant to control the development of bacteria—those causing food decomposition and above all pathogenic or potentially pathogenic bacteria for humans [19].

### 3.1. Physical Methods

One of the physical methods used to eliminate microbes from food is ionizing radiation. It was approved by the Food and Drug Administration as an acceptable food treatment method in the USA in 1997. However, this method is not fully effective and does not eliminate all bacteria. Moreover, it can adversely affect the appearance and other organoleptic attributes of certain food products, making them less attractive to consumers [20].

Another commonly used physical method for ensuring safe food is pasteurization, i.e., heat treatment in a temperature range from 65 to 75 °C in order to inactivate and reduce the number of bacteria [21]. This process is usually used for liquid products, such as fruit juice, beer, milk, and eggs. A similar method involving heat treatment is blanching, used in fruit and vegetable processing, in which the products are heated in order to deactivate enzymes causing the decomposition of food components. Sterilization is the process of inactivation of different forms (vegetative and spores) of microorganisms at a temperature more than 100 °C [22].

Pasteurization of fruits inactivates enzymes (pectinesterase and polygalacturonase) and destroys vegetative forms of spoilage bacteria, both Gram-negative and Gram-positive. During the pasteurization of beer, the wild yeasts and different Lactobacillus bacteria are inactivated. Milk pasteurization deactivates enzymes involved in spoilage and pathogenic or potentially pathogenic bacteria that may be present, e.g., *Salmonella*, *Brucella*, *Mycobacterium*, *Staphylococcus*, and *Streptococcus*. Pasteurization of eggs enables the deactivation of *Salmonella* bacteria [22].

Both pasteurization and high-pressure processing (sterilization) are not recommended for the preservation of fresh or meat products, because they can adversely affect the organoleptic properties of food and its nutritional value. Sterilization in high temperature ranges from 135 to 140 °C or 150 °C, and destroys all viable microorganisms including yeasts, molds, and vegetative form of bacteria as well as sporulating bacteria, resulting in a longer shelf life for food and at the same time a high degree of safety for consumers [23,24].

Among temperature-based methods, freezing, usually at a temperature of −18 °C to −30 °C, also plays a significant role, but has many shortcomings. First, the freezing process can cause degradation of food products; for example, many tropical fruits are highly sensitive to low temperatures. In some cases, organoleptic changes negatively affecting consumer acceptance may take place, e.g., the appearance of a gray or white coating due to crystallization of carbohydrates [22]. A safer process for products, especially those that are particularly sensitive to the effects of low temperatures, such as fruits, vegetables, and chicken eggs, is refrigeration, which does not significantly alter their organoleptic properties. However, because this method does not kill bacteria but only can inhibit the multiplication rate, an increase in the temperature has an impact on the multiplication of the remaining bacteria [22].

In many cases, high hydrostatic pressure (HPP) is used as a method of preservation and the reduction or inactivation of microorganisms. HPP reduces the number of cells of vegetative microorganisms causing food spoilage as well as pathogenic bacteria, which extends shelf life and improves the safety of food. Pressure treatment at 600 MPa causes deactivation of most vegetative forms of bacteria and fungal spores, but does not significantly inactivate bacterial spores, such as proteolytic spores of *Clostridium botulinum* [25]. The pressure applied for sterilization depends on the kind of food and potentially present microorganisms and usually ranges between 250 and 700 MPa (mainly 400 and 600 MPa), applied for a few seconds to 10 min [26]. The primary effects of HPP on bacterial cells are an increase in cell membrane permeability, disruption of protein structure and function, and finally, inhibition of the physiological activity of microorganisms [27]. HPP has been reported to cause morphological, structural, physiological, and genetic changes or damage to bacterial cells, e.g., *L. monocytogenes* [28]. HPP is often used to effectively reduce bacterial pathogens in liquid products as well as cooked, ready-to-eat food products [23,24]. HPP also delays the onset of chemical and enzymatic deteriorative processes by inactivating bacterial enzymes such as polyphenol oxidase and peroxidase. When HPP is used for animal products such as meat, it can induce changes in muscle enzymes (superoxide dismutase, catalase, and glutathione peroxidase), enhancing the antioxidant defense system and proteolysis rates and thereby improving the texture and structure of meat [28]. HPP can sterilize effectively, and due to the low processing temperature, can also protect natural components of food, including bioactive substances, especially in fruit and vegetable juices, as well as fresh fruit and vegetables [19]. In some cases, however, the use of high hydrostatic pressure for bio-preservation of fresh food may adversely affect the content of certain vitamins; for example, it can significantly reduce the concentration of ascorbic acid in fresh melons [24].

An interesting means of preservation, particularly of fresh fruits and vegetables, is the use of pulsed ultraviolet light (PUV). This is a non-thermal approach with high potential for decontamination of food, water, and air in food production environments [29]. This is achieved using ultra-short duration pulses of an intense broadband emission spectrum rich in UV-C light (200–280 nm band) of the highest germicidal efficacy. This UV spectrum mediates bacterial inactivation through several mechanisms, including damage to bacterial cells through the formation of pyrimidine dimers and the loss of cytoplasmic contents following light absorption [29,30]. The main bactericidal effect of UV-C light is caused by DNA damage. In addition, UV light has photophysical and photothermal effects on bacterial cells due to the absorption of the high-energy light pulses, resulting in leakage of the cell contents [29].

The efficacy of UV treatment in decontamination of food surfaces depends on many factors, such as the type of food, the distance between the product and the light source, the energy level, i.e., the number and frequency of light pulses, the duration of the treatment, the level of contamination, and others [29]. *L. monocytogenes* has been shown to be more resistant to UV-C light than other bacterial pathogens, such as *E. coli* [31]. The presence of organic materials such as food debris on stainless steel surfaces and NaCl content in ultraviolet-treated foods affect the efficacy of UV-C radiation on bacterial cells, due to the limited ability of the light to penetrate organic substances [32]. Other studies have also shown that the presence of salt in brine increases the amount of UV-C necessary to inactivate microbial pathogens in fluid [33].

UV radiation has proven effective at extending the shelf life of liquid egg white and various fruit juices, including apple, orange, pineapple, grape, cranberry, and pomegranate juice [34,35,36,37]. UV radiation reduces the number of bacteria in the food, but also increases the resistance of fresh food products like fruits and vegetables against bacterial contamination, especially Salmonella, Listeria, Staphylococcus, Clostridium, and Bacillus, and extends their shelf life [38,39].

The use of UV radiation for food preservation and safety depends on the type of microbes present in products, for which different doses and durations of UV-C radiation are applied. The recommended dose of UV radiation for inactivation of pathogens influencing food safety and resistance to decomposition is measured in mJ/cm^2^ and is 1–10 mJ/cm^2^ for bacteria, 2–8 mJ/cm^2^ for yeasts, 20–200 mJ/cm^2^ for other fungi, 100–150 mJ/cm^2^ for protozoa, and 300–400 mJ/cm^2^ for algae [36]. UV radiation has also been used for decontamination of the packaging of food products of both plant and animal origin, such as cartons, boxes, bottles, and trays [38].

### 3.2. Chemical Methods

Among other known food preservation methods, chemical preservation, involving the use of substances capable of inhibiting, retarding or arresting the growth of microbes, has by far the widest range of action against various species of bacteria and fungi [22]. Some of these food additives include “natural substances”, such as salt, sugar, vinegar or other acids, or rosemary extract. Artificial chemical preservatives include benzoic acid, sorbic acid, parabens, sulfur dioxide, sodium sulfite, and nitrate [22]. Chemical methods of preservation are used for conservation of both fresh and processed food; however, natural preservatives are most often used for bio-preservation of fresh food, like fish and fresh fruit, but also mayonnaise, margarine, and vegetable oils and fats [22]. Artificial preservatives are most often used in processed foods, such as butter, lard, meats, beer, wine, dried foods, baked goods, snacks, potato chips, nut products, powdered beverage mixes, sausages, and chicken broth. However, chemical preservatives can cause negative physical and chemical changes in food, reducing its biological value and causing undesirable effects on the body [22]. In many situations, the use of artificial preservatives has a negative effect on health, i.e., to the development of hyperactivity, loss of consciousness, cancer (e.g., nitrates), developing of asthma, and migraines (e.g., sulfites). Also, the disturbances in the concentration of bilirubin, urea, hemoglobin, and hematocrit are observed, especially in pregnant women, which can affect fetal development [22].

To control microbial contamination of fruits, vegetables, but also ready-to-eat foods, strong chlorine compounds or acids are used, mainly peracetic acid [39]. One of the antimicrobial agents used to preserve food is lactic acid, which is produced by lactic acid bacteria. Its role includes maintenance of the pH of the product, which inhibits the growth of bacteria that cause food contamination. For conservation of beef, pork, poultry, and fish, sodium lactate is used, which inhibits the growth of bacteria such as Clostridium spp., Yersinia spp., Listeria spp., Staphylococcus aureus, and E. coli. It has also been shown that simultaneous addition of sodium and calcium lactates yields a beneficial effect in inhibiting the growth of L. monocytogenes in seafood [39].

An interesting method of food bio-preservation is ozonation. Ozone is usually used as a method for water and sewage treatment, which is due to, among others, its physicochemical properties, which consist in decreasing solubility as the ambient temperature increases. Its mechanism of action on bacteria consists mainly in destroying protein structures responsible for transferring genetic material, including the cell membrane, capsid or envelope. This action limits the spread of microorganisms. The mechanism of bacterial cell membrane permeability disrupted by ozonation ultimately leads to the bacteria itself.

The ozonation method is also used for surface decontamination of fruits and vegetables and for combating insects and fungi present in cereals [19].

But, the negative consequences of using of chemical substances as preservatives are also connected to increased susceptibility to cancer and skin diseases, gastrointestinal, and multi-organ diseases and exacerbate the development of asthmatic processes [22,39]. Some may also have neurotoxic or embryotoxic effects. In many cases, stimulatory effects are observed as well. The substances used for preservation that cause DNA damage and mutations of the cells in organisms, like substances with E numbers (i.e., sodium benzoate E211; sulfur dioxide E220; sodium sulfite E221; potassium nitrite E249), seem to be particularly harmful [22].

### 3.3. Biological Methods

Another safe method for preserving food and reducing its levels of bacteria is fermentation, a biological method in which carbohydrates are broken down by microorganisms and/or enzymes. Bacteria, yeasts, and molds are most often used for fermentation of dairy, grain, and meat products [22]. Depending on the metabolic cycle and the microbes taking part in it, we can produce alcoholic, acetic acid or lactic acid fermentation.

Alcoholic fermentation is the result of the action of yeasts on simple hexose sugars, which are converted to alcohol and carbon dioxide. The quality of the fermented products depends on the presence of alcohol, which is an antibacterial agent and preservative in this system. Acetic acid fermentation takes place following alcoholic fermentation. During this fermentation, bacteria *Acetobacter* spp. convert alcohol to acetic acid by way of oxidation.

Lactic acid fermentation is mainly anaerobic respiration carried out by bacteria (*Lactobacillus* and others) to convert 3-carbon pyruvate to 3-carbon lactic acid (C_3_H_6_O_3_) and regenerate NAD+ in the process, allowing glycolysis to continue to produce ATP in low-oxygen conditions. This type of fermentation is one of the oldest and most commonly used methods of bio-preservation of various types of foods, i.e., in the production of fermented vegetables, fruits, fish, and traditional aged raw meat products [40,41].

Another type of fermentation involving probiotic strains is propionic fermentation, which produces propionic acid. Propionic acid bacteria (PAB) are a group of bacteria that ferment cheese and produce propionic acid [42]. The family *Propionibacteriaceae* consists of three genera: *Propionibacterium*, *Acidipropionibacterium*, and *Cutibacterium*. Some strains classified as PAB also produce bacteriocins that inhibit the growth of some bacteria. The metabolic activity of propionic acid provides other products as well, such as B vitamins (B_2_, B_12_, K, and folic acid) and nutrients, thus improving the stability and nutritional value of food products. However, propionic acid fermentation may negatively influence the growth of probiotic bacterial cells, e.g., during the formation of organic acids.

The resulting organic propionic acid has a very strong antibacterial effect on many Gram-positive and Gram-negative bacteria [43].

Examples of food preservation methods for selected food products are presented in Table 1.

### 3.4. Bacteriophages for Control of Bacteria in Food

Despite the significant development of various methods for detecting and eliminating foodborne pathogens at every stage of the food production process, in good production practices, quality control, and hygiene, as well as in the reorganization and improvement of animal rearing and agronomic processes, microbiological threats to food safety remain a common problem around the world. Another challenge in the food production sector is the need to limit or completely ban the use of certain antibiotics in animal production, due to the global problem of drug resistance of bacteria, and lack of new antimicrobial agents. Reducing the use of antibiotics in order to improve the quality and health of consumer products and limit the rise in drug resistance among pathogens is the subject of legal acts which are continually amended, e.g., in the European Union. This has resulted in the new EU Council Directive 2019/6, in force from 1 January 2022, which significantly limits the number of groups of antibiotics permitted for use in animal production [58].

Given the above, there is a great need to develop and implement alternative methods to antibiotics at the level of food production, guaranteeing adequate safety standards in terms of controlling foodborne pathogens and limiting their negative impact on people and animals [1].

One example of this method could be the use of bacteriophages, known as bacterial viruses, which are naturally occurring in the environment. Very promising results obtained at numerous research centers located all over the world have contributed to the introduction of many preparations containing bacteriophages to the commercial market. These are effective at controlling certain particularly important foodborne pathogens, including *L. monocytogenes*, *E. coli*, *Salmonella*, and *Campylobacter* [59,60]. Many of the currently used commercial preparations have obtained GRAS (Generally Recognized as Safe), as well as certificates from the U.S. Food and Drug Administration (FDA) for use in the United States and from Food Standards Australia New Zealand, including for elimination of pathogens from food of animal origin (meat products, milk, and dairy products) or plant origin (fresh fruits and vegetables) [61]. Some commercial phage preparations have been approved for use in EU countries to control food contamination (e.g., Listex^TM^ P100 and ListShield^TM^ for *L. monocytogenes* (Intralytix Inc., Baltimore, MD, USA) and as feed additives for food animals to control *S. enteritidis*, *S. typhimurium*, *E. coli*, and *L. monocytogenes* [62].

Bacteriophages are widespread in the natural environment, including in freshwater, where they number about 10^9^ phages/mL, and marine water—about 10^7^ phages/mL [63]. Bacteriophages can be a significant effective controlling bacteria that contaminate both fresh (meat, milk, vegetables, and fruits) and processed (cold cuts, ready-to-eat meals) food products, especially since they are safe and occur naturally in the environment. Their presence has been confirmed in fermented food, fresh vegetables, topsoil, and even delicatessen products, which means that both animals and humans are constantly exposed to bacteriophages or even ingest them [64].

Bacteriophages are able to independently attach to specific receptor sites on the surface of bacteria, and therefore they can infect only specific bacteria, while other cells or microorganisms present will be unaffected. This specificity for infection and lysis of target bacteria in combination with their prevalence in the environment, like water, soil, plants, animals, and sewage, is one of the key parameters for using phages in both detection and control of foodborne pathogens in food [1].

In the era of the development of organic food and growing awareness of healthy eating, natural food protection agents are becoming increasingly popular in production. Due to the high specificity of the action of bacteriophages, phage cocktails, which meet all the criteria to be recognized as ecological methods for controlling foodborne pathogenic bacteria, may play an important role [65]. The use of bacteriophages as a supplement to control microbial contamination of food also has many advantages. First, phages are highly specific and usually can infect only a single genus, species or even serotype of bacteria, which means that, in contrast to chemical compounds, they cannot destroy commensal microbiota in the digestive tract. Moreover, thus far they have not been shown to have any toxic effects on eukaryotic cells. Bacteriophages show a high resistance to factors arising during food processing, i.e., high temperatures and pressure, and as confirmed in many studies, phages do not have a negative influence on the sensory properties of food [65].

Many studies have also confirmed that bacteriophages can be used as potential tools for regulating the natural human intestinal microbiota [1]. According to Francino [66], bacteriophages selectively modify the intestinal microbiota, without causing dysbiosis. This is due in part to their prevalence in the environment of the digestive tract or to their production by intestinal commensal bacteria such as *E. coli*, containing prophage genetic material, which significantly reduces competition between different bacterial species colonizing the gut [67]. Bacteriophages are currently the subject of research at many research centers all over the world, including in Poland. Apart from therapeutic aspects, this work has enabled assessment of their effectiveness against bacteria present in food [1]. For this reason, bacteriophages are used as additives in both commercial and experimental preparations to extend the shelf life of many food products of both animal and plant origin (Table 2).

Apart from the demonstrated benefits of using bacteriophages as potential antibacterial agents, there are a number of factors that may limit their use as food bio-preservatives. For example, bacteria are capable of developing resistance to some phages, and the antibacterial efficacy of phages is lower than that of conventional sterilization techniques. In addition, there are major problems with adjusting the specific storage and use conditions for phages, such as a sufficiently low temperature and pH range appropriate for a given phage. An important negative effect is the phage titer decreasing with longer storage times. Because of the protein structure of capsid, bacteriophages may induce an immune response in the body. In addition, contamination of food, e.g., dairy products and silage, with bacteriophages can be a serious problem in the fermentation industry. A very important factor that may potentially limit the use of bacteriophages in food bio-preservation is the fact that consumers may be unwilling to accept the presence of bacterial viruses in food [91].

## 4. Conclusions

The increase in the global human population is the main cause of the increased demand for food meeting standards of biological safety, shelf life, and organoleptic attributes in line with consumer needs. Programs for controlling pathogenic bacteria in the food supply chain, especially at the primary production stage, play a significant role in the production of food which is safe for consumers, as one of the main priorities of the European Union and other countries in the world. On the other hand, a number of methods—chemical, physical and biological—have been developed for effective decontamination of food which has already been produced. This food is also safe, although some of the means of reducing or eliminating microorganisms may have unwanted effects, such as adverse organoleptic changes or consumer unwillingness to consume food treated in this manner.

The development of natural and ecological food preservation methods seems to be a current priority in the food production sector. It is difficult to say whether the methods used in the future will be completely new or modernizations of methods already used today. Continued development of research on the use of natural and ecological methods for reducing microbial contamination, based on bacteriophages specific for pathogens, can significantly contribute to the production of even safer food with a longer shelf life.

## Figures and Tables

**Table 1:** Examples of food preservation methods from current literature.

Classification of Method	Conditions	Type of Foods	Effect	References
Physical				
Non-thermal				
UV irradiation	UV 1–400 mJ/cm^2^	Fresh fruits, vegetables; fruit juices—apple, orange, pineapple, grape, cranberry, pomegranate	Antimicrobial activity Inhibition of food decomposition, preservation	[38]
Ionizing radiation (IOR)	Radioactive cobalt-60 or high-penetrating cesium-137; X-ray 5 MeV; high-energy electron accelerators ≤10 MeV	Surface of agri-foods, equipment packaging, work surfaces in food production	Inactivation of microbes	[44]
Cold plasma (non-thermal plasma)	Combination of ions, UV photons, electrons, reactive species, and charged elements	Milk and dairy products, beef, poultry, wheat grain	Inactivation of microbes through destruction of structural elements of cells	[45,46,47,48]
Pulsed electric field (PEF)	10–80 kV/cm/s	Juices—apple, orange, tomato, carrot; applesauce, salad dressing, pea soup, eggs, milk and dairy products	Enhancement of physicochemical, rheological, and antioxidant properties	[49]
High-pressure processing (HPP)	100–800 MPa/temp. <20 °C for a few seconds to 1 min	Fruits, meat, vegetables, milk and their products, drinks, seafood, fish	Inactivation of a variety of pathogenic and spoilage vegetative bacteria, yeasts, molds, viruses, and spores; prevention of sensory changes	[50]
Ultrasound technology	16–100 kHz 100 kHz–10 MHz	Milk and dairy products, apples, potatoes	Microbicidal effect Unfavorable changes in the structure of products	[51]
Pulsed light (PL)	200–1000 nm	Processing of liquid and solid food, e.g., fish, vegetables, fruit, and meat	Inactivation of microbes Change in the conformation of food allergens due to protein aggregation	[52]
Thermal Refrigeration (cooling/chilling)	2 °C	Plant products, dairy products, eggs	Inhibition of microbial development and enzyme activity; extension of shelf life	[53]
	−2 °C	Meat products, coffee	Inhibition of multiplication of microorganisms	[22]
	−1 to 8 °C	Dairy products, vegetables, fruits, meat, fish, ready-to-eat dishes	Slowing down decomposition, extension of shelf life	
Freezing	−18 °C to −30 °C	Vegetables, fruits, mushrooms, meat, fish	Inhibition of multiplication rate, extension of shelf life	[53]
Pasteurization	65 °C for 30 min; 77 °C for 1 min; 80 °C for 10–60 s	Fruit juice, milk products, beer, liquid eggs	Destruction of vegetative forms of microorganisms, inactivation of bacterial enzymes causing food spoilage	[22]
Blanching	70–100 °C/1–15 min	Fruits and vegetables	Destruction of enzymes contributing to food decomposition	[53]
Heat sterilization	>100–150 °C	Milk, ready-to-eat processed meat and vegetable products	Destruction of microbes, inactivation of enzymes causing decomposition, extension of shelf life	[22,25]
Drying	45–70 °C 30–70 °C 4–70 °C 80 °C	Fruits Vegetables Fish Meat Instant coffee, tea	Inhibition of bacterial and fungal growth	[22]
Biological				
Fermentation Homofermentative (30–40 °C) and heterofermentative (15–22 °C) strains		Bread, wine, beer, vinegar, cocoa, coffee Yogurt, cheese, sauerkraut	Inhibition of multiplication of bacteria and fungi	[54,55]
Lactic acid bacteria: *Lactococcus*, *Streptococcus*, *Lactobacillus*, *Pediococcus*, *Leuconostoc*, *Enterococcus*, *Carnobacterium*, *Aerococcus*, *Oenococcus*, *Tetragenococcus*, *Vagococcus*, and *Weisella* *Acetobacter* spp.	Lactic acid Ethanol, Acetic acid, ethanol, and carbon dioxide	Dairy products; cream or yogurt-based products, cheese, meat, fruits, and vegetables, including sauerkraut Cereals, and fruits and vegetablesBeer, wine, vegetables, sourdough bread Yogurt, cheese, vinegar, and sauerkraut	Control of pH, increased competition through multiplication of probiotic strains	[56]
*Pediococcus cerevisiae*	Lactic and acetic acid	Vegetables: cucumbers, bell pepper, olives; salami	Inhibition of mold, some yeasts and Gram-negative bacteria	[43,54,56]
*Propionibacterium* spp. *Acidipropionibacterium* spp., Yeast: *Saccharomyces cerevisiae*	Propionic acid fermentation Alcohol fermentation	Cheese, dairy by-products Bread, wine, beer, vinegar, cocoa, coffee		
Chemical				
pH control	6.0–6.8 4.1–5.3 4.0–4.4 4.4–4.6 2.8–3.5 4.0–5.8 4.5–5.0 2.5–5.5 4.6–6.4	Milk, cheese, fruit yogurts, plain yogurt, wine, baked bread, marmalades, syrups, fresh fruit, vegetables		[22]
Natural preservatives: salt, sugar, vinegar, rosemary extract	-	Fish, vegetables, jams, confitures, vegetables, oils, and fats	Inhibition of decomposition processes; extension of shelf life	[22]
Artificial preservatives	Sulfates, nitrites and benzoates; emulsifers, stabilizers and thickeners; anticaking agents; preservatives; leavening agents; flavoring agents; coloring agents; fat replacers	Wine, beer, dried fruits		[22,57]
Ozone treatment	Combined sp2 and 2p2 orbitals, which brings about dual 9-molecular orbitals	Disinfection of fruit and vegetable surfaces Disinfection of drinking water Sewage treatment Processing of meat and seafood Control of insects/fungi in grain storage	Inactivation of microbes by destroying the structure of protein and genetic material; food preservation	[19]
Other Vacuum packaging	(a) Steam-sterilized metal cans (b) Paper, films and laminates sterilized with hot hydrogen peroxide (c) Plastic and metal containers sterilized with steam under high pressure	Meat, milk, processed foods	Reduction in microbial contamination; extension of shelf life	[19]

**Table 2:** Examples of phage efficacy to control various bacteria at various stages of food production.

Foodborne Pathogens	Type of Phage Cocktail	Food Product	Company or Experimental Product; Certification	Results	References
*Salmonella* spp., *E. coli*, *Listeria monocytogenes*, *Shigella* spp.	Ecolicide PX™	Beef, poultry, fruits, vegetables, dairy products (including cheese), fish, other seafood	Intralytix, Inc.; (Baltimore, MD, USA) GRAS (GRN No. 000834)	Significant reduction (3 log_10_ CFU/g) in *E. coli* O157:H7 on the skin of living animals	[68,69]
*E. coli* O157:H7 O45:H2 O103:H2 O111:H- O121:H19	Cocktail of four phages specific for STEC	Beef	Experimental phage cocktail	Reduction in the number of *E. coli* bacteria by 0.4–0.7 log_10_ CFU/cm^2^ on pieces of cowhide Variation was shown in the susceptibility of *E. coli* to the phages used	[70]
*E. coli* O157:H7 STEC	EcoShield™ (ECP-100); Phage cocktail	Vegetables	Intralytix, Inc.; FDA	Reduction by 1–3 log_10_ CFU/g or below the limit of detection on tomatoes, broccoli or spinach	[71]
	EcoShield PX™ Phage cocktail	Poultry and beef products, fish, cheese, vegetables	Intralytix, Inc.; FDA, FCN No. 1018	Reduction in bacteria by 3.0 log CFU/g) in ground beef, chicken breast, boiled chicken, salmon, cheese, cantaloupe, and romaine lettuce Significant ≥80% reduction in *E. coli* O157:H7 on roast beef with neck	[69]
*E. coli* O157	PhageGuard E	Meat and poultry, sprayed on whole heads or packaged leafy vegetables—cut green salads, lettuce; pet food	PhageGuard; FDA and USDA, the Netherlands	Reduction in *E. coli* O157 up to 99.9% on leafy greens Reduction in bacteria up to 3-log_10_ CFU on leafy vegetables	[72,73]
*E. coli*	Secure Shield E1	Beef products, turkey, and other foods	FINK TEC; GmbH (GER) FDA/GRN 724, USDA, FSIS Directive 7120.1		[74]
EPEC *E. coli* Nmr-2	Cocktail with five phages: K EPEC, BI EPEC, BL EPEC, CI EPEC, and CS EPEC 10^6^ CFU/m	Shrimp, chicken meat, milk, lettuce, tofu	Experimental phage cocktails	Reduction in the number of EPEC by 0.24 log_10_ CFU on lettuce and 1.84 log_10_ in milk No reduction in bacteria in tofu, chicken meat or shrimp	[75]
*Salmonella* spp.	PhageGuard S *Salmonella* phages S16 and FO1a	Ground and coarse beef and poultry meat; pet food	PhageGuard.com; FDA, EU, Canada, Australia, New Zealand, Switzerland, Israel	Reduction in *Salmonella* of about > 1–2.4 log Kill of *Salmonella* (90–99.6%), log_10_ CFU	[72]
*Salmonella* Enteritidis	Phage solution sall_v01 approx. 10^7^ PFU/mL	Pig slurry	Application of phage sall_v01	Significant reduction in *S. enteritidis* bacteria by 3.8 log_10_ CFU/mL in pig slurry	[76]
*Salmonella* Enteritidis	Phage mixture (SCPLX-1) of four phages titer 3 × 10^8^ PFU/mL	Fresh melon	Experimental solution of *Salmonella* phage SCPLX-1	Reduction by 2.5 log_10_ CFU at 20 °C and 3.5 log_10_ CFU at 10 °C from melon	[77]
*Salmonella enterica* ser. Typhimurium; Enteritidis; Pullorum; Dublin	LPSTLL, LPST94, LPST153 phage cocktail MOI of 1000 (add 10 µL of 8 log_10_ PFU/mL phage to reach a final titer of 6 log_10_ PFU/mL) or 10,000 (add 10 µL of 9 log_10_ PFU/mL phage to reach a final titer of 7 log_10_ PFU/mL) in milk Phage cocktail was added with an MOI of 1000 (spot 10 µL of 8 log_10_ PFU/mL phage to reach a final titer of 6 log_10_ PFU/cm^2^) or MOI of 10,000 (spot 10 µL of 9 log_10_ PFU/mL phage for a final titer of 7 log_10_ PFU/cm^2^) by pipette transferring the lysate followed by spreading the lysate with a sterile spreader on surface of chicken breast samples	Chicken, meat, milk		3.0 log reduction in *Salmonella* inoculum to below detectable limits on chicken breast and in milk Phage LPST153 lysed 50–100% strains of nine *Salmonella* serovars (except two serovars; Newport and Kentucky) Phage cocktail was effective against *Salmonella* biofilm grown for 72 h on microtiter plates and steel chips, resulting in >5.23 log reduction in *Salmonella* viable cells	[4,78]
*Salmonella enterica* ser. Typhymurium Typhi Ty 2-b Paratyphi	Cocktail of three phages, BSPM4, BSP101, BSP22A at titer ~1 × 10^8^ PFU/mL	Lettuce and cucumber		Reduction of 3.9 log_10_ CFU of *S. Typhimurium* numbers on lettuce and 2.8 log reduction on cucumber after 4 h incubation at 25 °C	[79]
*Salmonella* Enteritidis, Typhimurium, Paratyphi A, San Diego, and Typhi	Cocktail of four *Salmonella* phages (CAU-SEP-1, CAU-SEP-2, CAU-SEP-3, and CAU-SEP-4) titer 10^8^ PFU/mL	Chicken breast meat	Experimental phage cocktail	Decrease in the bacteria count up to 3.12 log CFU/mL after 6 h treatment	[80]
*Salmonella* Enteritidis	Bacteriophage LSE7621 titer (~10^8^ PFU/mL)	Lettuce, tofu	Experimental phage solution	For tofu—significantly reduced bacteria by 3.55 log_10_ CFU/mL For lettuce—reduction by 1.02 log_10_ CFU/mL	[81]
*Salmonella enterica* ser. Typhimurium, Enteritidis, Heidelberg, Newport, Hadar, Kentucky, Thompson, Georgia, Agona, Grampian, Senftenberg, Alachua, Infantis, Reading, and Schwarzengrund	SalmoFresh™	Poultry, fish, and shellfish, fresh and processed fruits and vegetables Ready-to-eat processed products	Bacteriophage cocktail concentrations of ≥10^8^ PFU/mL; GRN No. 435,Canada	Greater than 4.0-log_10_ CFU/mL reductions of pathogens for vegetables; Average of 5.34 log_10_ CFU/mL after 5 h at 25 °C after spraying SalmoFresh™ onto lettuce and sprouts Inactivated more than 90% of the *Salmonella* population (10^1^ to 10^3^ CFU/mL)	[82,83,84]
*Shigella flexneri*	Bacteriophage vB_SflS-ISF001 (10^8^ PFU/g)	Cooked and raw chicken breast	Experimental phage cocktail	Reduction ~2 logs_10_ CFU/mL of *Shigella* numbers within the first 24 h in cooked and raw chicken breast	[85]
*Shigella* spp., including *S. sonnei*	ShigaShield™ (2 × 10^7^ or 9 × 10^7^ PFU/g)	Deli meat, smoked salmon, pre-cooked chicken, melons, lettuce, yogurt	Five-phage GRAS-affirmed cocktail(GRN 672)	Reduced levels of *Shigella* by approx. 1 log_10_ CFU/mL	[86]
*Campylobacter jejuni*	Cocktail of two *Campylobacter* phages (F356, F357)	Chicken meat and skin	Experimental phage cocktail	Reduction in *Campylobacter* strains 0.55–0.79 log_10_ CFU/mL	[87]
*Campylobacter jejuni*	Phage Cj6 titer 10^4^ PFU/g-cm^2^	Phage applied to raw and cooked contaminated beef products Skin stored at 5 °C or 24 °C for up to 8 days	Experimental phage	Significant bacteria inactivation—3 log_10_ cm^−2^ at 5 °C and 45.9 log_10_ cm^−2^ in beef products	[88]
*Campylobacter* spp. (*C. jejuni. C. coli*)		Raw red meat (including whole carcasses, primal and subprimal cuts, trimmings, and organs), and raw poultry; raw beef slices	FDA: GRN 966 USA	Reduction in *Campylobacter* spp. 1–3 log_10_ CFU/mL on artificially contaminated cooked and raw beef slices Reduction in bacterial content about 0.7 log_10_ CFU/mL on chicken skin	[69]
*Listeria* spp.	PhageGuard L.	Ready-to-eat meats (fermented pork sausage, dry cured ham); cooked turkey and roast beef; fresh or frozen fruits and vegetables	PhageGuard; FDA	Reduction in *Listeria* >1 log kill by scientific researchers on various ready-to-eat meats Reduction in bacteria up to 2 log_10_ CFU from fresh or frozen vegetables	[72]
*Clostridium perfringens*	Phage cocktail *C. perfringens* JCM1290	Chicken meat, milk	Experimental phage *C. perfringens* JCM1290T suspension (5 × 10^7^ PFU/mL)	Decrease in bacteria in pasteurized milk by 0.4 log_10_ CFU at 24 °C Decreased bacteria count in chicken meat by 1.2–2.5 log CFU/piece	[89]
*R. anatipestifer*	PhagePharm (CHN)	Aquaculture environments	JiangYanQing; FDA, FSIS China	Decrease in bacteria in aquaculture	[90]

Legend: GRAS—Generally Recognized as Safe; FCN—Food Contact Notification; FSIS—Food Safety and Inspection Service; N/A—Not available.

## Data Availability

No new data were created or analyzed in this study. Data sharing is not applicable to this article.
